# Evaluation of keratometric and total corneal astigmatism measurements from optical biometers and anterior segment tomographers and mapping to reconstructed corneal astigmatism vector components

**DOI:** 10.1371/journal.pone.0313574

**Published:** 2025-01-08

**Authors:** Achim Langenbucher, Nóra Szentmáry, Alan Cayless, Peter Hoffmann, Jascha Wendelstein, Seth Pantanelli

**Affiliations:** 1 Department of Experimental Ophthalmology, Saarland University, Homburg/Saar, Germany; 2 Dr. Rolf M. Schwiete Center for Limbal Stem Cell and Aniridia Research, Saarland University, Homburg/Saar, Germany; 3 Department of Ophthalmology, Semmelweis-University, Budapest, Hungary; 4 School of Physical Sciences, The Open University, Milton Keynes, United Kingdom; 5 Augen- und Laserklinik Castrop-Rauxel, Castrop-Rauxel, Germany; 6 Department of Ophthalmology, Ludwig-Maximilian-University, Munich, Germany; 7 Department of Ophthalmology, Pennsylvania State University, Hershey, PA, United States of America; Chiemsee Augen Tagesklinik, Technical University of Munich, GERMANY

## Abstract

**Purpose:**

To investigate different measures for corneal astigmatism in the context of reconstructed corneal astigmatism (recCP) as required to correct the pseudophakic eye, and to derive prediction models to map measured corneal astigmatism to recCP.

**Methods:**

Retrospective single centre study of 509 eyes of 509 cataract patients with monofocal (MX60P) IOL. Corneal power measured with the IOLMaster 700 keratometry (IOLMK), and Galilei G4 keratometry (GK), total corneal power (TCP2), and Alpin’s integrated front (CorT) and total corneal power (CorTTP). Feedforward shallow neural network (NET) and linear regression (REG) prediction models were derived to map the measured C0 and C45 power vector components to the respective recCP components.

**Results:**

Both the NET and REG models showed superior performance compared to a constant model correcting the centroid error. The mean squared prediction errors for the NET/REG models were: 0.21/0.33 dpt for IOLMK, 0.23/0.36 dpt for GK, 0.24/0.35 for TCP2, 0.23/0.39 dpt for CorT and 0.22/0.36 dpt for CorTTP respectively (training data) and 0.27/0.37 dpt for IOLMK, 0.26/0.37 dpt for GK, 0.38/0.42 dpt for TCP2, 0.35/0.36 dpt for CorT, and 0.44/0.45 dpt for CorTTP respectively on the test data. Crossvalidation with model optimisation on the training (and validation) data and performance check on the test data showed a slight overfitting especially with the NET models.

**Conclusions:**

Measurement modalities for corneal astigmatism do not yield consistent results. On training data the NET models performed systematically better, but on the test data REG showed similar performance to NET with the advantage of easier implementation.

## Background

Calculation of toric intraocular lenses (tIOL) requires a reliable description of the corneal astigmatism to be corrected with the implant [1–7]. Manual keratometers, automated keratometers (e.g. keratometers integrated in an optical biometer), and corneal (Placido-based) topography all measure the curvature of the corneal front surface. Whereas manual keratometers typically measure at 2 points each at the flat and steep corneal meridian, automatic keratometers integrated in an optical biometer either measure at multiple points or derive keratometry from a Placido-pattern (SimK values). The keratometric values are typically taken in the mid-periphery at a diameter of 2.0 to 3.6 mm depending on the hardware setting and the curvature of the cornea.

While the mean corneal front and back surface curvatures normally exhibit some degree of correlation [8–11], this is not true for the flat and steep or horizontal and vertical meridians [12, 13]: the corneal back surface measurement is typically more curved in the vertical meridian and less curved in the horizontal meridian, which adds some astigmatism against-the-rule (axis of the flat meridian in a range between 60 and 120 degrees) to the cornea. This means that corneal astigmatism with-the-rule (axis of the steep meridian in a range between 60 and 120 degrees) is overestimated and corneal astigmatism against-the-rule is underestimated by a keratometer [14, 15].

Even though Javal’s rule [16] as modified by Grosvenor [14, 17] may reflect the average in a larger population, the impact of corneal back surface astigmatism may vary between individual eyes, and the disparity between refractive cylinder and keratometric astigmatism may also include non-corneal components such as tilt or decentration of the crystalline lens [14, 16].

Modern tomographers are capable of measuring the curvature of both corneal surfaces, making a ‘thick lens model’ description of the cornea possible [1, 4, 6, 12, 18, 19]. However, there are still some concerns about the precision of the corneal back surface measurements [7, 11, 20, 21], and the description of the cornea in terms of 2 surfaces and crossed cylinders might be cumbersome in clinical practice. Therefore, clinicians often use nomograms or regression corrections to transform keratometric astigmatism into the total corneal astigmatism used for tIOL power calculation [7, 12, 20]. Most of these nomograms are based on a vector decomposition of corneal astigmatism and spectacle cylinder, where the bivariate astigmatism given in terms of an absolute cylinder value and axis is converted to a bivariate vector composed of the projection of the astigmatism to the 0/90 degree meridian (C0) and the projection to the 45/135 degree meridian (C45). These vector components are mostly displayed using double angle plots where C0 is plotted on the X-axis and C45 is plotted on the Y-axis [22, 23].

With the crystalline lens in place we cannot properly separate corneal astigmatism and lens astigmatism, as phakometry is not really reliable. However, after cataract surgery with implantation of stigmatic or toric intraocular lens (IOL) it is possible to derive the optical vergence in front of the corneal front vertex plane from the pseudophakic spectacle refraction and behind the corneal front vertex plane from the IOL power (IOLP) and position (ELP) and the axial length (AL). The vergence difference (vergence behind minus vergence in front of corneal front vertex plane) reconstructs the corneal power recCP in terms of equivalent power (.)_EQ_ and astigmatism (.)_AST_. Since this reconstructed corneal power is based on the refraction and IOL power, it automatically incorporates any effects of surgically-induced astigmatism and thus presents an advantage over other concepts if it can be accurately predicted from preoperative keratometry.

The **purposes of the present study** were

- using keratometric and total corneal astigmatism values derived from an optical biometer and an anterior segment tomographer commonly used for tIOL calculation, to investigate their effects on reconstructed corneal astigmatism
- to derive recCP from pseudophakic spectacle refraction and biometry and IOLP,
- to decompose astigmatism of measurements and recCP into vector components and compare measurements with the reconstruction, and
- to derive shallow feed-forward neural network and multivariate regressions to map the measured corneal astigmatism vector components to the respective components of recCP.

## Methods

### Dataset for our study and surgical details

A dataset with N = 509 clinical data points (from N = 509 patients) Department of Ophthalmology, Penn State College of Medicine (Hershey, PA, USA) was considered for this retrospective study. All data were anonymised at source and stored in a.XLSX file, which was transferred to the Department of Experimental Ophthalmology for further analysis (February 03, 2024). Data tables were reduced to the relevant parameters required for our analysis, consisting of patient age in years, gender, laterality of the eye (OS or OD), refractive power of the intraocular lens (IOLP in dpt) and the measured parameters from the following devices:

IOLMaster 700 (Carl-Zeiss-Meditec, Jena, Germany):

The following indices were measured using an IOLMaster 700 (Carl-Zeiss-Meditec, Jena, Germany): Axial length (AL) in mm, anterior chamber depth (ACD) in mm (considered from the corneal epithelium to the front apex of the crystalline lens), central thickness of the crystalline lens (LT) in mm, horizontal corneal diameter (CD) in mm, and keratometry (IOLMK).

Galilei G4 (Ziemer Ophthalmic Systems, Brügg, Switzerland):

Keratometry (GK) in the flat meridian (in dpt) together with the axis of the flat meridian (in degrees) and in the steep meridian (in dpt), total corneal power (GTCP2) centred on the pupil in the flat meridian(in dpt) together with the axis of the flat meridian (in degrees) and in the steep meridian (in dpt), area integrated corneal front surface power (CorT [24, 25]) in the flat meridian (in dpt) together with the axis of the flat meridian (in degrees) and in the steep meridian (in dpt), and area integrated total corneal power (CorTTP) in the flat meridian (in dpt) together with the axis of the flat meridian (in degrees) and in the steep meridian (in dpt). CorT and CorTTP were evaluated using Alpin’s method by means of a separate software package (ASSORT) installed on the Galilei G4 [24, 25]. According to Alpin’s method, CorT and CorTTP are derived by calculating the astigmatism in different ring zones and then combining the ring astigmatism values via vector summation.

Refraction:

Manual refraction (REF) with sphere (in dpt) and cylinder (in dpt) at the refractive cylinder axis (in degrees) measured with a phoropter at a refraction lane distance of 6 m.

Eyes with missing or incomplete data in any of the above mentioned values were excluded at the source. All eyes were measured before cataract surgery with the IOLMaster 700 and Galilei G4 and at least 21 days postoperatively with manual refraction.

All surgeries were performed or supervised by an experienced surgeon (SP) under topical anaesthesia. After para-limbal 2.4 mm micro incision from the temporal side the anterior chamber was filled with a dispersive OVD, and a continuous curvilinear capsulorhexis slightly smaller than the IOL optic diameter (approximately 5.5 mm) was created. Following a standard phacoemulsification procedure, the MX60P IOL (Bausch & Lomb, Vaughan, Ontario, Canada) was inserted, taking special care that all viscoelastic behind and surrounding the IOL was removed and the continuous curvilinear capsulorhexis and paracenteses were hydrated. Since the dataset was completely anonymised, the Institutional Review Board considered this to be non-human subjects research and was therefore exempt from review (Ärztekammer des Saarlandes, 157/21). Informed consent of the patients was not required. The study followed the tenets of the Declaration of Helsinki.

### Pre-processing of the data

The data were transferred to Matlab (Matlab 2022b, MathWorks, Natick, USA) for further processing. Custom software was written in Matlab to decompose IOLMK, GK, TCP2, CorT, CorTTP, and REF from standard notation into power vector components in terms of (spherical) equivalent power (.)EQ and the astigmatism projected to the 0/90 degree meridians (.)C0, and astigmatism projected to the 45/135 degree meridians (.)C45 [22, 23]. The defocus equivalent DEQ was derived from the power vector components of the spectacle refraction using DEQ = (REFEQ^2^ + 1/4·REFC0^2^ + 1/4·REFC45^2^)^1/2^. To account for lateral symmetry of the power vectors, the power vector components for the oblique axis (.)C45 were flipped in sign for all left eyes to consider all eyes as right eyes [3, 8].

### Reconstruction of the astigmatism of corneal spherocylindrical power recCP

For this calculation we assume a simplified pseudophakic eye model with 3 refracting surfaces and the focal plane at AL behind the cornea: a thin lens (spherocylindrical) spectacle refraction at the vertex distance of 12 mm in front of the cornea, a (spherocylindrical) thin lens cornea, and a thin lens IOL at an effective lens position (ELP) behind the cornea. For the refractive indices we used n = 1.0 for air and n_A_ = n_V_ = 1.336 for the aqueous and vitreous humour [26]. The ELP was derived according to the Haigis formula [27] based on a linear regression with an intercept a_0_ and weighting a_1_ for ACD and a_2_ for AL, and the optimised formula constants listed in IOLCon (www.IOLCon.org, accessed on April 14, 2024). For the MX60P we used a_0_/a_1_/a_2_ = 0.1835/0.3153/0.1725.

Assuming the retina to be at the focal plane, the vergence at the ELP plane was V3_ = (AL-ELP)/n_V_. Considering the IOL of refractive power IOLP the vergence directly in front of the IOL is V3 = V3_—IOLP. When transferred to the corneal front vertex plane this becomes V2_ = V3/(1-V3·ELP/n_A_) [3, 9, 12, 13]. Assuming a vergence V1 = -1/6 dpt directly in front of the spectacle plane (with a lane distance of 6 m), the vergence behind the spectacle plane V1_ is derived by considering the spherocylindrical refraction (vector components REFEQ, REFC0 and REFC45). Tracing V1_ through the vertex distance we obtain the spherocylindrical vergence V2 directly in front of the corneal front surface plane. The reconstructed corneal power recCP is calculated as the difference between the vector components of V2_ and V2, respectively [3, 9, 12, 13].

### Prediction models to map measured corneal power to recCP

The astigmatism vector components ((.)C0 and (.)C45) were used to define prediction models to map the measured astigmatism components of IOLMK, GK, TCP2, CorT, and CorTTP to the respective components of recCP. Data were split randomly into a training set (N = 305, 60%), validation set (N = 102, 20%) and a test set (N = 102, 20%). For the shallow feedforward neural network (NET) with 2 hidden layers and 12 nodes per layer we used the training set during the learning process and for fitting the weights [3, 9, 12]. The validation set is used to tune the hyperparameters and to control potential overfitting, and the test set is then used as an independent dataset for assessing the prediction performance [9].

For simple clinical use we also defined bivariate linear regression based prediction models (REG) to map the vector components of the measured corneal astigmatism to the recCP vector components. We used maximum likelihood estimation with iterative ECM algorithm [28, 29], and the respective results are described in terms of LogL as the value of the log likelihood objective function after the final iteration.

The terms NETIOLMK / REGIOLMK, NETGK / REGGK, NETTCP2 / REGTCP2, NETCorT / REGCorT, and NETCorTTP / REGCorTTP describe the mapping of IOLMK, GK, TCP2, CorT, CorTTP to recCP respectively using a shallow feedforward neural network / bivariate linear regression approach.

### Statistical analysis and data presentation

Data were listed exploratively using the arithmetic mean, standard deviation (SD), median, and the lower and upper boundaries of the 95% confidence interval (2.5% and 97.5% quantiles). The astigmatic power vector components C0 and C45 were analysed using polar double angle plots showing the C0 / C45 vector component in the horizontal / vertical axis [22, 23]. As a simplification, assuming bivariate normal distributions for the C0 / C45 vector, error ellipses for the 95% confidence intervals were calculated from the variance-covariance matrices, and the centroids and areas of the error ellipses (derived from the eigenvalues and eigenvectors [3, 9]) were documented. The mean length of the vector difference (MVD) as derived from the distribution of the vector difference length was reported as a clinical measure, and the mean squared prediction error (MSE) was used as a generally accepted metric [21] to evaluate the differences between reconstructed power recCP and measurements IOLMK, GK, TCP2, CorT, CorTTP, and performance for the 5 regression based and feedforward neural network based prediction models separately for the training and the test data.

## Results

All 509 eyes were treated as independent cases (265 female and 181 male, 261 OS and 248 OD). The mean patient age was 70.52 ± 11.23 years (median 72 years). In **Table 1** the most relevant explorative data for the N = 509 eyes are listed in terms of mean, standard deviation, median, and the bounds of the 95% confidence interval for biometric measures AL, ACD, LT, CD, IOLP, and postoperative refraction REFEQ and REFC.

**Table 1:** Explorative listing of most relevant preoperative biometric measurements axial length AL, anterior chamber depth ACD (measured from the corneal epithelium to the front apex of the crystalline lens), thickness of the crystalline lens LT, and horizontal corneal diameter CD as derived with the IOLMaster 700, power of the implanted lens IOLP, postoperative refraction (spherical equivalent REFEQ, cylinder REFC and defocus equivalent DEQ), and the effective lens position ELP predicted with the Haigis formula. Mean, SD, Median, and 2.5% / 97.5% refer to the arithmetic mean, standard deviation, median, and the lower and upper bounds of the 95% confidence interval respectively.

N = 509	AL in mm	ACD in mm	LT in mm	CD in mm	IOLP in dpt	REFEQ in dpt	REFC in dpt	DEQ in dpt	ELP in mm
Mean	24.03	3.32	4.58	12.02	20.30	-0.25	0.64	0.60	5.37
SD	1.45	1.53	0.46	0.55	4.01	0.62	0.59	0.53	0.58
Median	23.79	3.22	4.58	12.00	21.00	-0.25	0.50	0.53	5.31
2.5% quantile	21.98	2.50	3.62	11.29	10.00	-2.17	0.00	0.00	4.85
97.5% quantile	27.71	4.05	5.410	12.90	27.00	0.75	2.25	2.26	6.16

**Table 2** lists the descriptive data for the astigmatic power vector components C0 and C45 for IOLMK, GK, TCP2, CorT, CorTTP and the reconstructed corneal power recTP together with the area of the error ellipse indicating the 95% confidence region. The X and Y coordinates of the centroids are directly described by the mean values of the C0 and C45 astigmatic vector components.

**Table 2:** Astigmatic vector components (projections to the 0/90 degree meridian (C0) and to the 45/135 degree meridian (C45)) for keratometry with the IOLMaster 700, and keratometry, TCP, and integrated corneal front surface power CorT and total corneal power CorTTP of the Galilei G4 ^3,4^ together with the astigmatism of the reconstructed corneal power recCP. Mean, SD, Median, and 2.5% / 97.5% refer to the arithmetic mean, standard deviation, median, and the lower and upper bounds of the 95% confidence interval respectively. The mean value of the C0 / C45 component is equivalent to the coordinates of the centroids. The last row shows the area (in dpt^2^) of the error ellipse indicating the 95% confidence region.

N = 509; data in dpt	IOLMaster 700		Galilei G4								Reconstructed corneal power recCP	
	Keratometry		Keratometry		TCP2 mm		CorT		CorTTP			
Component	C0	C45	C0	C45	C0	C45	C0	C45	C0	C45	C0	C45
Mean	0.0046	-0.0598	0.1394	-0.1235	-0.1407	-0.1420	0.0135	-0.0272	-0.1278	-0.1293	-0.3149	-0.0498
SD	0.8585	0.5341	0.8088	0.5167	0.8764	0.5362	0.7336	0.4277	0.8734	0.5118	0.6706	0.4482
Median	0.0230	-0.0670	0.1042	-0.1372	-0.1761	-0.1599	-0.0350	-0.0202	-0.1465	-0.1373	-0.1586	0.0000
2.5% quantile	-1.7802	-1.1204	-1.4149	-1.1661	-1.6809	-1.2049	-1.4006	-0.9115	-1.7563	-1.0927	-1.9518	-1.0995
97.5% quantile	1.8326	1.0030	1.7059	0.8183	1.6951	0.9415	1.3624	0.8568	1.4632	1.0008	0.9598	0.9024
95% error ellipse area in dpt^2^	8.6110		7.8287		8.8333		5.9039		8.4136		5.6259	

The upper part of **Table 3** shows the definitions of the bivariate linear regression based and feedforward shallow neural network based prediction models which map the vector components C0 and C45 derived from IOLMaster 700 keratometry (REGIOLMK and NETIOLMK), Galilei G4 keratometry (REGGK and NETGK) total corneal power (REGTCP2 and NETTCP2), integrated front surface (REGCorT and NETCorT) and total corneal power (REGCorTTP and NETCorTTP) to the respective vector components of the reconstructed corneal power recCP derived from the N = 305 training dataset. In the lower part of the table, the X coordinates (for the C0 component) and Y coordinates (for the C45 component) of the centroids, the areas of the error ellipses indicating the 95% confidence region, the mean vectors difference MVD as a clinical measure, and the mean squared prediction error MSE, are displayed for the difference recCP minus measured corneal power IOLMK, GK, TCP2, CorT, CorTTP and for the 5 regression based and 5 feedforward neural network based prediction models separated for the training data and the test data. As expected the centroid coordinates for the regression based models equal zero for the training data. The prediction performances (in terms of MSE) for NETIOLMK, NETGK, NETTCP2, NETCorT and NETCorTT are systematically better for the training data and similar for the test data compared to REGIOLMK, REGGK, REGTCP2, REGCorT and REGCorTTP. Especially the NET prediction models, but also the REG prediction models show some overfitting with higher MSE values and larger error ellipses in the test data compared to training data. Both the NET and REG prediction models show a systematically better performance in terms of MSE and the areas of the error ellipses compared to recCP minus measured corneal power values for the training and test data, and the smaller areas of the error ellipses for the NET and REG prediction models compared to recCP minus measured corneal power values indicate that the NET and REG prediction models outperform a constant model with a centroid correction only.

**Table 3:** Upper part: Definitions of the bivariate linear regression based and feedforward shallow neural network based prediction models which map the vector components C0 and C45 of IOLMaster 700 keratometry (REGIOLMK and NETIOLMK), Galilei G4 keratometry (REGGK and NETGK), total corneal power (REGTCP2 and NETTCP2), integrated front surface (REGCorT and NETCorT) and total corneal power (REGCorTTP and NETCorTTP) to the respective vector components of the reconstructed corneal power recCP. The entire dataset was split into training, validation, and test sets (60%/20%/20%), and the training data were used to define the prediction model. Lower part: Listing of the centroid X- and Y-coordinates in dpt, area of the error ellipse indicating the 95% confidence region in dpt^2^, mean vector difference MVD in dpt, and mean squared prediction error MSE in dpt^2^ for recCP–measured corneal power, the regression based, and the feedforward neural network based prediction models separated for the training data and the test data. As expected, the regression based models yield centroid coordinates at the origin for the training data. The prediction performance (in terms of MSE and area of the error ellipses) for NETIOLMK, NETGK, NETTCP2, NETCorT and NETCorTTP are slightly better mostly for the training data compared to REGIOLMK, REGGK, REGTCP2, REGCorTP and REGCorTTP, and both NET and REG models reduce the difference recCP-meaqsured corneal power systematically. Both the regression and feedforward models show a slight overfitting with higher MSE values in the test data compared to training data.

N = 509		Equation of the bivariate linear prediction model										logL	
Data in dpt													
Prediction model:													
Linear regression model from N = 305 training data	REGIOLMK	${\left[\begin{array}{c} recCPC0\\ recCPC45 \end{array}\right]}_{\mathrm{predicted}}=\left[\begin{array}{cc} 0.543 & 0.070\\ -0.01 & 0.375 \end{array}\right]∙\left[\begin{array}{c} IOLMKC0\\ IOLMKC45 \end{array}\right]+\left[\begin{array}{c} -0.304\\ -0.043 \end{array}\right]$										-332	
	REGGK	${\left[\begin{array}{c} recCPC0\\ recCPC45 \end{array}\right]}_{\mathrm{predicted}}=\left[\begin{array}{cc} 0.524 & 0.010\\ -0.024 & 0.336 \end{array}\right]∙\left[\begin{array}{c} PK0\\ PKC45 \end{array}\right]+\left[\begin{array}{c} -0.392\\ -0.023 \end{array}\right]$										-337	
	REGTCP2	${\left[\begin{array}{c} recCPC0\\ recCPC45 \end{array}\right]}_{\mathrm{predicted}}=\left[\begin{array}{cc} 0.508 & -0.051\\ -0.002 & 0.269 \end{array}\right]∙\left[\begin{array}{c} PTCRP2C0\\ PTCRP2C45 \end{array}\right]+\left[\begin{array}{c} -0.252\\ -0.021 \end{array}\right]$										-277	
	REGCorT	${\left[\begin{array}{c} recCPC0\\ recCPC45 \end{array}\right]}_{\mathrm{predicted}}=\left[\begin{array}{cc} 0.559 & -0.088\\ 0.038 & 0.314 \end{array}\right]∙\left[\begin{array}{c} CorTC0\\ CorTC45 \end{array}\right]+\left[\begin{array}{c} -0.302\\ -0.053 \end{array}\right]$										-276	
	REGCorTTP	${\left[\begin{array}{c} recCPC0\\ recCPC45 \end{array}\right]}_{\mathrm{predicted}}=\left[\begin{array}{cc} 0.512 & -0.006\\ 0.022 & 0.313 \end{array}\right]∙\left[\begin{array}{c} CorTTPC0\\ CorTTPC45 \end{array}\right]+\left[\begin{array}{c} -0.236\\ -0.024 \end{array}\right]$										-257	
recCP-measured corneal power & Model prediction error in dpt		Training data (N = 305)					Test data (N = 102)						
		Centroid X	Centroid Y	Error ellipse area in dpt^2^	MVD	MSE		Centroid X	Centroid Y	Error ellipse area in dpt^2^	MVD		MSE
recCP-measurement	recCP-IOLMK	-0.2897	-0.0046	5.5670	0.7035	0.6873		-0.3864	0.1021	5.6606	0.7704		0.8443
	recCP-GK	-0.4353	0.0434	6.0857	0.7958	0.8588		-0.5251	0.1464	5.2413	0.8430		0.8554
	recCP-TCP2	-0.1690	0.0599	6.6274	0.7240	0.7514		-0.1887	0.1437	7.4968	0.7848		0.8967
	recCP-CorT	-0.2760	-0.0523	5.7083	0.7141	0.6999		-0.4010	0.0275	4.8162	0.7124		0.6680
	recCP-CorTTP	-0.1414	0.0318	6.4595	0.7219	0.7202		-0.2131	0.1757	7.0445	0.7627		0.9238
Linear regression model	REGIOLMK	0.0000	0.0000	3.0218	0.3715	0.3290		-0.0380	0.1046	3.1226	0.4438		0.3708
	REGGK	0.0000	0.0000	3.2861	0.4020	0.3619		-0.0452	0.0935	3.2720	0.4341		0.3683
	REGTCP2	0.0000	0.0000	3.2290	0.4073	0.3539		-0.0054	0.0629	3.7714	0.4692		0.4173
	REGCorT	0.0000	0.0000	3.5766	0.4025	0.3943		-0.0526	0.0662	3.3660	0.4490		0.3616
	REGCorTTP	0.0000	0.0000	3.2831	0.3873	0.3595		-0.0190	0.0891	3.9854	0.4782		0.4478
Feedforward neural network model	NETIOLMK	-0.0195	-0.0007	1.8955	0.4664	0.2057		-0.0454	0.0893	2.2998	0.5240		0.2694
	NETGK	0.0223	0.0166	2.0901	0.5051	0.2307		-0.0344	0.0844	2.3374	0.5164		0.2626
	NETTCP2	0.0095	0.0438	2.1293	0.4943	0.2356		0.0104	0.0898	3.2467	0.5314		0.3753
	NETCorT	0.0046	0.0031	2.0909	0.5241	0.2287		-0.0407	0.0671	3.1697	0.4912		0.3514
	NETCorTTP	0.0110	-0.0184	1.9763	0.4981	0.2226		0.0234	0.0342	3.7455	0.5393		0.4390

**Fig 1:**
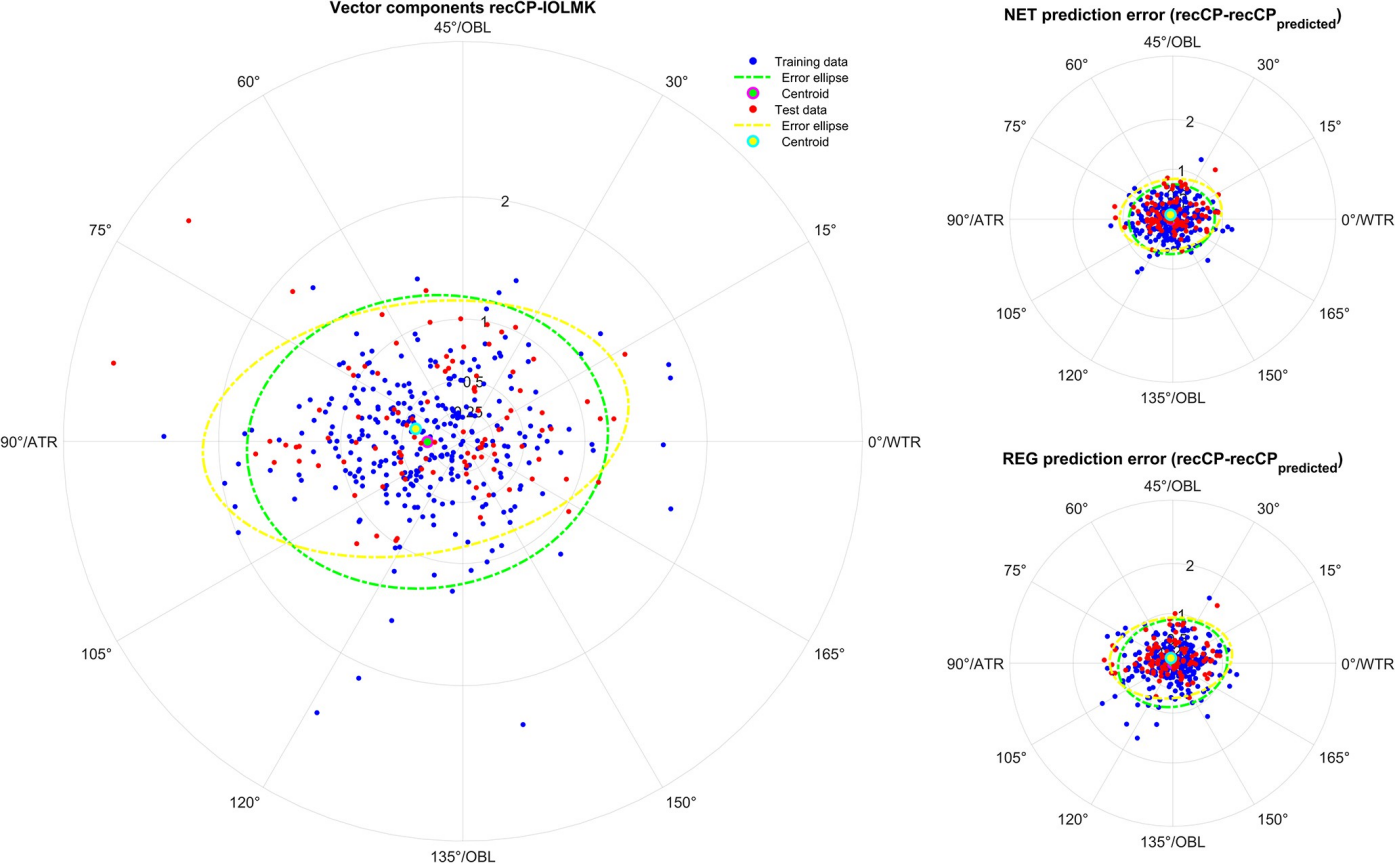
Double angle plots for the keratometry measures of the Zeiss IOLMaster 700 device showing the 2 astigmatic power vector components (C0 and C45: Projection to the 0/90 degree and to the 45/135 degree meridian) for the differences between recCP and measured corneal power (graphs on the left) and the model prediction errors (prediction error: recCP—recCP_predicted_) for the feedforward neural network based models (NET: Upper graphs on the right) and the linear regression based models (REG: Lower graphs on the right) for different measures modalities. The data (blue dots referring to the training data and red dots referring to the test data) are shown together with the 95% error ellipses (green and yellow dash-dotted lines) and the centroids (green and yellow filled circle markers) for the training dataset (N = 305) and the test dataset (N = 102).

**Fig 2:**
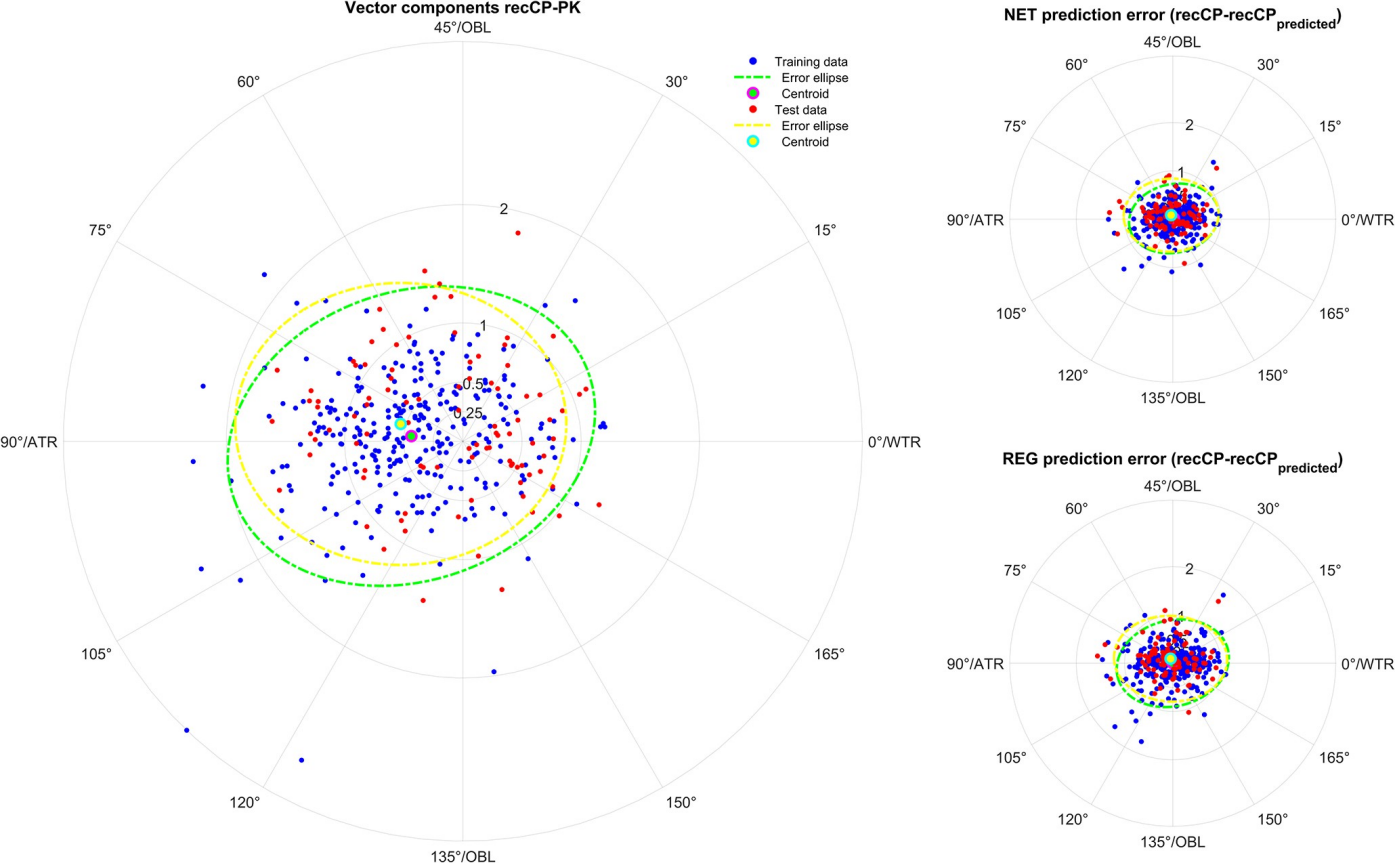
Double angle plots for the keratometry measures of the Ziemer Galilei G4 device (GK) showing the 2 astigmatic power vector components (C0 and C45: Projection to the 0/90 degree and to the 45/135 degree meridian) for the differences between recCP and measured corneal power (graphs on the left) and the model prediction errors (prediction error: recCP—recCP_predicted_) for the feedforward neural network based models (NET: Upper graphs on the right) and the linear regression based models (REG: Lower graphs on the right) for different measures modalities. The data (blue dots referring to the training data and red dots referring to the test data) are shown together with the 95% error ellipses (green and yellow dash-dotted lines) and the centroids (green and yellow filled circle markers) for the training dataset (N = 305) and the test dataset (N = 102).

**Fig 3:**
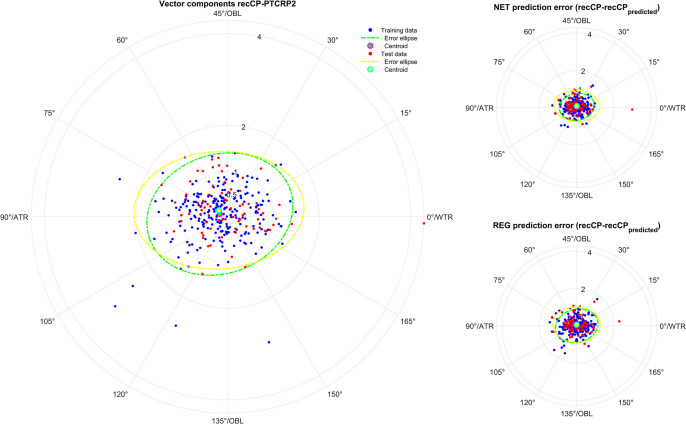
Double angle plots for the total corneal power values of the Ziemer Galilei G4 device (TCP2) showing the 2 astigmatic power vector components (C0 and C45: Projection to the 0/90 degree and to the 45/135 degree meridian) for the differences between recCP and measured corneal power (graphs on the left) and the model prediction errors (prediction error: recCP—recCP_predicted_) for the feedforward neural network based models (NET: Upper graphs on the right) and the linear regression based models (REG: Lower graphs on the right) for different measures modalities. The data (blue dots referring to the training data and red dots referring to the test data) are shown together with the 95% error ellipses (green and yellow dash-dotted lines) and the centroids (green and yellow filled circle markers) for the training dataset (N = 305) and the test dataset (N = 102).

**Fig 4:**
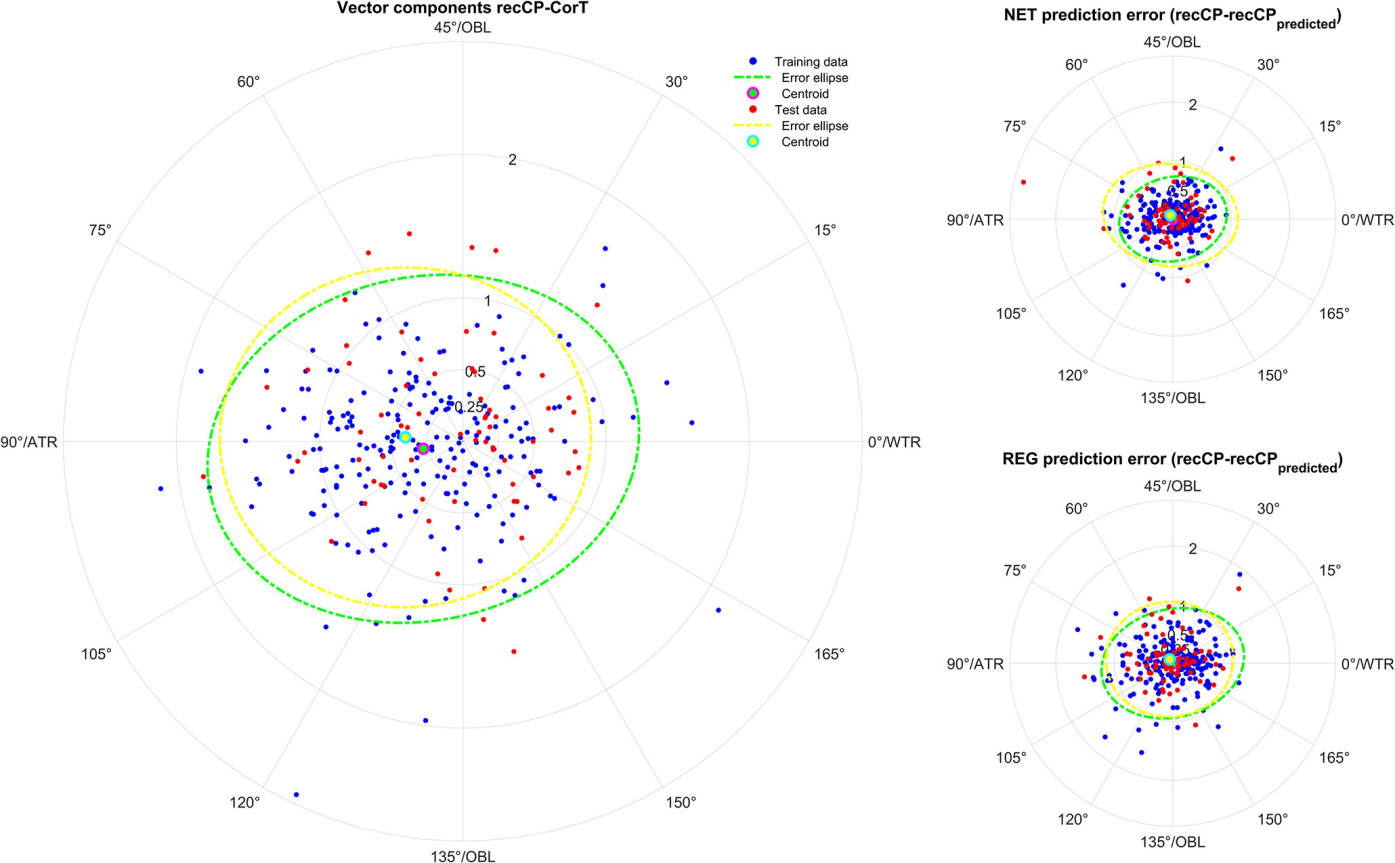
Double angle plots for the integrated corneal front surface power measures of the Galilei G4 device (CorT) according to the Alpin’s method showing the 2 astigmatic power vector components (C0 and C45: Projection to the 0/90 degree and to the 45/135 degree meridian) for the differences between recCP and measured corneal power (graphs on the left) and the model prediction errors (prediction error: recCP—recCP_predicted_) for the feedforward neural network based models (NET: Upper graphs on the right) and the linear regression based models (REG: Lower graphs on the right) for different measures modalities. The data (blue dots referring to the training data and red dots referring to the test data) are shown together with the 95% error ellipses (green and yellow dash-dotted lines) and the centroids (green and yellow filled circle markers) for the training dataset (N = 305) and the test dataset (N = 102).

**Fig 5:**
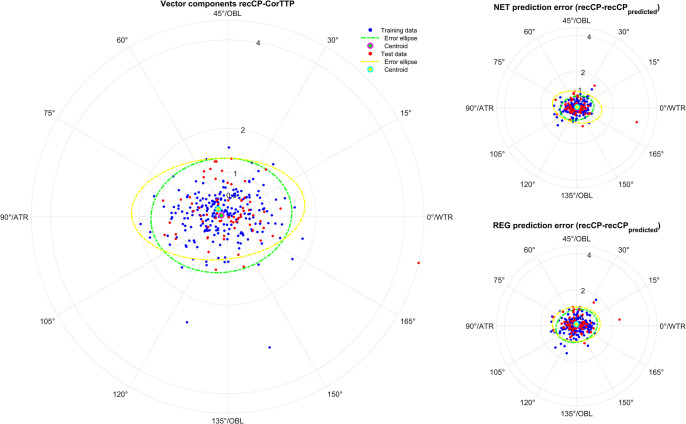
Double angle plots for the integrated total corneal power measures of the Galilei G4 device (CorTTP) according to the Alpin’s method showing the 2 astigmatic power vector components (C0 and C45: Projection to the 0/90 degree and to the 45/135 degree meridian) for the differences between recCP and measured corneal power (graphs on the left) and the model prediction errors (prediction error: recCP—recCP_predicted_) for the feedforward neural network based models (NET: Upper graphs on the right) and the linear regression based models (REG: Lower graphs on the right) for different measures modalities. The data (blue dots referring to the training data and red dots referring to the test data) are shown together with the 95% error ellipses (green and yellow dash-dotted lines) and the centroids (green and yellow filled circle markers) for the training dataset (N = 305) and the test dataset (N = 102).

**Figs 1–5** displays polar double angle plots showing the astigmatic power vector components for the reconstructed–measured corneal power (graphs on the left) together with the model prediction errors for the NET prediction models (upper right graphs) and the REG prediction models (lower right graphs) for different corneal astigmatism measurement modalities. The plots depict IOLMK **(Fig 1)**, GK **(Fig 2)**, TCP2 **(Fig 3)**, CorT **(Fig 4)**, and CorTTP **(Fig 5)**. The blue and the red dots refer to the coordinates of the N = 305 training and N = 102 test data. The error ellipses (green and yellow dash-dotted lines) together with the filled circle markers (green and yellow) refer to the 95% error ellipses and the centroids of the bivariate distributions for the training data and the test data. The respective areas of the ellipses are listed in the lower part of **Table 3**.

## Discussion

Since Javal’s basic work on astigmatism more than 100 years ago it is known that the corneal front surface astigmatism does not properly represent the entire astigmatism of the eye [14, 16, 17]. in addition to astigmatic effects from the slanted visual axis and decentration and tilt of the crystalline lens, the corneal back surface has a significant impact on the mismatch between corneal front surface astigmatism and total astigmatism of the eye [5, 13]. Currently, and especially for calculation of toric IOLs, the keratometric values can be used with or without any nomogram or regression correction or alternatively both corneal surfaces can be measured with a corneal or anterior segment tomographer to obtain the total corneal astigmatism [7, 20]. However, even though some modern optical biometers do offer the option of measuring the corneal back surface mean curvature and astigmatism, others are restricted to measuring the corneal curvature at 6 or more distinct points or topographically with a projection of a Placido pattern.

Several correction strategies have been published, all of which systematically add some astigmatism against-the-rule to the corneal front surface astigmatism [1, 2, 6, 10, 15, 17, 18, 28, 30–32]. Today we know that Javal’s rule [14] with or without Grosvenor’s modification [14, 17] is a simplification which can potentially overestimate the impact of the corneal back surface astigmatism. We have to distinguish between correction strategies which aim to map keratometric astigmatism to the total corneal astigmatism derived from a tomographer, and strategies which aim to map keratometric astigmatism to corneal astigmatism retrieved from spectacle refraction after cataract surgery [7, 20]. The first option is restricted to the impact of the corneal back surface, whereas the second option also considers potential effects of a slanted visual axis and tilt and decentration of optical elements in addition to the corneal back surface astigmatism [1, 2, 18, 30, 31].

In the current paper we have back calculated the corneal power and astigmatism from the spherocylindrical spectacle refraction after cataract surgery with implantation of a non-toric IOL together with the ELP and the IOLP using vergence transformation. This corneal power was used as the standard / target for our correction strategy. Based on measurements from modern optical biometers such as the IOLMaster 700 and the Galilei G4 we developed simple feedforward shallow neural network with 2 hidden layers and robust bivariate linear regression prediction models [3, 9, 12] to map the IOLMK, GK, TCP2, CorT, and CorTTP [24, 25] to the astigmatism of recCP. Given the symmetry in astigmatism axis between left and right eyes [8], we decided to consider all eyes as right eyes by flipping the C45 component of refraction and corneal power in sign. To properly evaluate the performance of the prediction models and a potential overfitting, the entire dataset was randomly split into subsets of training data, validation data, and test data with a ratio of 60% / 20% / 20%. The training data were used to establish the NET (weights and biases) and REG prediction models. The validation data were then used with the NET prediction models to provide an unbiased evaluation of the model fit on the training data while tuning the hyperparameters. Finally, the test data were used to provide an unbiased evaluation of the final NET and REG prediction models on the training dataset.

The current study builds on a previous study in which we used a calculation strategy based on 3D power vectors (with equivalent power and the 2 astigmatic power vector components) to derive the reconstructed corneal power from spherocylindrical refraction after cataract surgery and the labelled power together with the measured orientation of the toric lens [33]. In this previous paper we used a dataset with N = 442 eyes which included preoperative and postoperative keratometry and Total Keratometry readings made using the IOLMaster 700.The implanted lenses used in this previous study were the monofocal or multifocal toric intraocular lenses ZeissTORBI and LISA. The present study extends this work by applying a similar approach, but this time using a 2D vector representation, to a new dataset of N = 509 eyes measured using both the IOLMaster 700 and the Galilei G4, and implanted with a different lens, the Bausch & Lomb MX60P IOL.

The results from the previous study are consistent with our findings from the current new study, both showing that the neural network approach outperforms the classical multilinear regression on the training data, but with a strict crossvalidation the prediction performance of the neural network and the multilinear regression were quite similar [33]. This demonstrates that the approach outlined previously has wider applicability to other datasets and lens types.

Our results in **Table 2** show a large variation in the mean C0 and C45 values comparing the measurements. For example, with IOLMK and CorT the mean C0 component is -0.06 dpt whereas the same component is around 0.14 dpt with GK and -0.13 / -0.14 with TCP2 and CorTTP respectively. In comparison, the mean C0 of recCP is around -0.31 dpt. For the C45 component the IOLMK and CorT provide mean values close to 0 (-0.06 and -0.03 dpt) whereas other measures give systematically more negative values (-0.12 / -0.14 / -0.13 dpt for GK / TCP2 / CorTTP). In comparison, the mean C45 of recCP is around -0.05 dpt.

This implies that if we intend to correct the centroid error only [34] (using a constant model) we should add -0.32 / 0.01 dpt to the C0 / C45 component for IOLMK, -0.47 / 0.07 dpt to GK, -0.18 / 0.08 dpt to TCP2, -0.32 / -0.04 to CorT, and -0.18 / 0.07 to CorTTP. C0 centroid corrections are much larger for the corneal front surface measures IOLMK, GK, CorT than for the total corneal power measures TCP2 and CorTTP, which is consistent with the literature [1, 2, 4–7, 18, 20, 30–32].

From the definitions of the linear regression prediction models REG shown in **Table 3** we learn that a simple centroid correction with a constant model is not the best solution. The values on the main diagonal which give the translation of the measured to the predicted C0 (element (1,1)) and C45 component (element (2,2)) are all systematically smaller than 1, meaning that for large (positive or negative) C0 or C45 components measured with IOLMK, GK, TCP2, CorT and CorTTP the respective predicted recCP C0 or C45 value is smaller. The lower part of this table shows that the areas of the error ellipses and also the MSE are systematically larger for the difference between the reconstructed and measured corneal power values (recCP–IOLMK / GK / TCP2 / CorT / CorTTP) compared to the respective areas and MSE values for the NET and REG prediction models. This means that both prediction model architectures show a good performance, and the smaller areas of the error ellipses for NET and REG prediction errors compared to recCP–measured corneal power again underlines that a simple centroid correction with a constant model (which simply shifts the entire distribution to the origin by letting the area of the error ellipse unchanged) might not be the best solution.

As expected, all REG prediction models fully correct the centroid error on the training data [9]. In contrast the NET prediction models which aim to minimise the MSE for the training data do not zero the centroid error. On the test data neither the REG nor the NET prediction models exactly zero the centroid error. However, our results for the training data show that the NET prediction models produce error ellipses of smaller area, and smaller MSE values, than the REG prediction models. This is not surprising as the NET models are capable of adapting more flexibly to the data structure even in case of large nonlinearities. However, for the test data, which is essential for a proper cross-validation, the NET prediction models no longer systematically outperform the REG prediction models and both the areas of the error ellipses and the MSE are mostly similar. Furthermore, with both the NET and REG prediction models we observed some overfitting, with both the areas of the error ellipses and our performance metric MSE being systematically larger for the test data compared to the training data [9]. This means that without cross-validation the performance of our prediction models is strictly overestimated.

Comparing the graphs on the right of **Figs 1–5** with the corresponding graphs on the left shows that the prediction error both with REG and NET is surprisingly low compared to the difference between reconstructed and measured corneal power (the scales on the axes are identical for all graphs in **Figs 1–5**). This means that with the NET or REG correction we strictly gain performance in predicting recCP astigmatism. In particular, comparing the sizes of the error ellipses for the NET and REG model prediction errors to the respective sizes on the left graphs shows that all of the prediction models outperform a centroid correction (using a constant model), which simply shifts the distributions with the centroids to the origin without change in the error ellipse area.

Even though the REG prediction models show very slightly inferior performance compared to the NET prediction models on the test data, the implementation in any common office PC software (e.g. Excel) is much simpler, consisting of the following steps:

1. decompose the measured corneal power or astigmatism into C0 and C45 vector components,
2. consider all eyes as right eyes by flipping the sign of the C45 vector components for left eyes [8],
3. apply the respective prediction model as shown in the upper part of **Table 3**, and
4. convert back the predicted corneal power vector components to standard notation (cylinder magnitude and axis) after flipping the sign of the C45 vector component for left eyes.

However, our study has some limitations: firstly, we back-calculated the corneal power recCP from the refraction data after cataract surgery. Since pseudophakic refraction also includes optical aberrations (e.g. astigmatism or coma) resulting from a slanted visual axis or decentration and tilt of the IOL, recCP may not properly represent the total corneal astigmatism. Secondly, all implanted IOL were non-toric and this might cause a selection bias towards lower corneal astigmatism since eyes with a larger pre-existing corneal astigmatism would be more likely to receive toric lens implants. Finally, motivated by previous papers we assumed symmetry between left and right eyes [8] and ‘mirrored’ left eyes by flipping the sign of the C45 component to be considered as right eyes. With this technique we could define a common model for left and right eyes, but the parameters may be slightly different if separate prediction models for left and right eyes were used.

**In conclusion**, our results indicate that both feedforward shallow neural network prediction models and linear regression models show excellent performance in predicting the total corneal astigmatism as required for toric intraocular lens power calculation, with the neural network models performing slightly better in some situations. Both types of model strictly outperform simple centroid corrections (in terms of constant models) which simply shift the entire distributions with its centroid to the origin. However, the total corneal astigmatism cannot be predicted perfectly based on the measurement of the IOLMaster 700 or Galilei G4 values, and this implies that a direct measurement of both corneal surfaces might be the best option if corneal or anterior segment tomography is available. Further multicentric studies with larger populations and including toric and non-toric lenses should be performed to further prove the validity of these prediction models.
